# What Drives Outpatient Care Costs in Kenya? An Analysis With Generalized Estimating Equations

**DOI:** 10.3389/fpubh.2021.648465

**Published:** 2021-09-22

**Authors:** Ngugi Mwenda, Ruth Nduati, Mathew Kosgei, Gregory Kerich

**Affiliations:** ^1^School of Aerospace and Physical Science, Department of Mathematics, Physics and Computing, Moi University, Eldoret, Kenya; ^2^Department of Pediatrics, University of Nairobi, Nairobi, Kenya

**Keywords:** GEE, outpatient, healthcare, *QIC*_*u*_, cost

## Abstract

**Objective:** This study aimed to identify the factors associated with outpatient expenses incurred by households in Kenya.

**Background:** The problem of outpatient healthcare expenses incurred by citizens in countries with limited resources has received little attention. Thus, this study aimed to determine the predictors of household spending on outpatient expenses in Kenya.

**Method:** We conducted a cross-sectional analysis on households in Kenya using data from the 2018 Kenya Household Health Expenditure and Utilization Survey. We applied the generalized estimating equations method to determine the best subset of predictors of outpatient care cost.

**Findings:** The best predictors of outpatient care expenses in Kenya are age, wealth index, and education level of the household head.

**Conclusions:** There were no differences regarding age in the mean spending on outpatient care. Moreover, we found that the cost of outpatient care changes with age in a sinusoidal manner. We observed that rich households spent more on outpatient care, mostly owing to their financial ability. Households whose heads reported primary or secondary school education level spent less on outpatient costs than households headed by those who never went to school.

## Introduction

Although Kenya is a lower middle-income country (LMIC), it is one of the fastest growing economies in sub-Saharan Africa (1). To ensure steady economic growth and proper social development, the need has emerged to stabilize the national health systems of Kenya (2).

Although the country continues to strive toward reforming its healthcare system, it faces challenges in the form of financial constraints, high debt (3), a high debt-to-gross domestic product ratio of 70%, weak institutional capacity, and a high unemployment rate [almost 20% (4)], which in turn raises the dependency ratio (5). Thus, there are significant obstacles to effective change. Owing to a constrained budget, funds allocated to the healthcare sector remain low (6). The recent budget allocation of 9.1% to the healthcare sector as a proportion of total government budget (7) is low; this is contrary to the 2001 Abuja Declaration on healthcare in Africa that at least 15% of the budget be allocated to the healthcare sector. Therefore, to achieve any substantial advancement, Kenya's health sector requires comprehensive improvements or even complete reformation (2).

Owing to limited availability of resources in LMICs (8), sound and accurate evidence is needed to formulate and implement health policies that are influenced by the current state of a country's economy (9). Still, the presence of evidence might not be sufficient for the prioritization of resource allocation, given the existence of other more demanding factors, such as political strategies and donor demands on funding (10). Therefore, developing countries have to make difficult choices about how to allocate limited resources and spending with a view to maximizing their output (11).

By contrast, primary healthcare in developed countries has continuously benefited from medical security policies, provided proper medical care to citizens, alleviated the economic burden of disease by reducing catastrophic spending on health, and provided financial support to ease the burden of healthcare by making current data available (12–17).

According to the existing literature, demand for inpatient and outpatient healthcare is likely to increase in the next decade owing to population growth outpacing growth in the supply of health facilities (18). This increased demand is due to cardiovascular diseases, obesity (19), and respiratory illnesses, like COVID-19 (20).

With limited resources due to reduced revenue collection by the government as a result of falling household incomes (21), the need to re-allocate resources gives rise to opportunity costs, leading to gains in one sector and losses in another. This exerts pressure on the governments of developing countries, which have limited resource availability, to take decisions to meet the expected increase in demand.

Prior studies have established that more than 11 million Africans, of whom 0.45 million are Kenyans, are pushed into extreme poverty every year because of out-of-pocket and outpatient health expenses (22). To create awareness about this healthcare spending strain, the Kenyan government has made consistent efforts to insure significantly more of the population through the National Health Insurance Fund (NHIF); however, 83% of the Kenya population of 50 million were uninsured as of 2017 (23).

Measures have been taken to reform the NHIF (24), which could be Kenya's gateway to achieving universal health coverage (UHC) (25). This was accomplished by conducting a pilot study in a few counties (Nyeri, Kisumu, Machakos, and Isiolo), where the state was to meet all the medical costs (26, 27) and advance toward achieving Sustainable Development Goal (SDG) 3 (28). The purpose of the pilot study was to determine the possibility and sustainability of implementing the program in the entire country.

The pilot study is in line with the ongoing global drive toward attaining UHC in LMICs, which has paved the way for health sector reforms to help realize this objective. The main objective of UHC is to cushion citizens against the catastrophic and impoverishing effects of out-of-pocket healthcare payments, such as those in Kenya (22, 29), which have led to household poverty (30), socio-economic inequality, inequity in the use of healthcare services (31), and time wastage from traveling long distances to access healthcare services (2). Unfortunately, when analyzed in the global context, Kenya's achievements remain inadequate (26). The findings from the pilot study indicate that tax revenues collected by the Kenyan government are not sufficient to fully support UHC.

Household spending on outpatient care is an important characteristic of financial expenditure for measuring public health (23). However, existing studies have not focused on this issue, because certain health conditions might not expose the affected to risk. However, it is important to note that if proper medical attention is not provided, some health conditions that may appear insignificant can easily deteriorate with time. Thus, household spending is key to arresting the deteriorating condition of outpatients.

The choice of seeking outpatient care when sick or injured could be influenced by (1) the seriousness of the health condition of the affected person and (2) the person's financial ability to pay for the required healthcare services (32). In this case, key determinants are the characteristics of the household figure or care provider from whom the household member needs to seek help (33–35).

Most often, the head of the household is the breadwinner and makes vital decisions in the household. The education level of the household head in a study in Uganda determined whether birth delivery took place in the presence of a skilled birth attendant (36). A study in Nicaragua showed that in some households, even though the woman earned more, decisions regarding the household, including expenditure, were made by the male household head (37). A study in Nepal found that, although women were involved in decision making in the household, they did not have autonomy with respect to final decisions, as the man was regarded as the household head (38). Although most households regard the man as the household head, this is not always the case, as recent literature in Kenya shows that about 36% of households are female headed. Another study in South Africa indicates that heads have final say over decisions regarding household expenditure, even when they do not earn the most income (39).

Given the tendency of the household head in Africa to influence the members of a household, it is imperative to investigate outpatient care predictors with reference to the characteristics of the household head.

## Materials and Methods

### Study Design and Population

The data were collected through a cross-sectional study carried out from April 9, 2018 to May 19, 2018 across all 47 counties in Kenya, called the Kenya Household Health Utilization Survey (KHHEUS). The survey was household based and designed to provide estimates for various indicators at the national, residence (urban and rural), and county levels. The sample design constituted 1,500 clusters with 923 rural and 577 urban residences spread across the country. The sampling consisted of two stages: the first was a stratified cluster sampling design in which 1,500 clusters were selected, and the second was a uniform sample of 25 households, which were randomly selected.

A questionnaire designed by a technical working group of the Kenya National Bureau of Statistics (KNBS), World Bank (WB), and Ministry of Health (MoH) was administered to every sampled household, and it was pretested, reviewed, and improved before the training. More information on the questionnaire design can be found elsewhere (40).

Briefly, the KHHEUS questionnaire objectives were set by the MoH, WB, and KNBS officials. Its main aim is to determine household expenditure on health services used for both inpatient and outpatient care. It is the collective responsibility of the technical working group to develop and review the survey tools, recruit and train enumerators, collect data, write reports, and disseminate them.

The questionnaire collected information on the utilization of outpatient and inpatient services. Other details collected that were useful for this study included household composition, health insurance, housing conditions, assets, amenities, household consumption, and expenditure.

The training was organized in three levels. First, the trainers were trained between March 13 and 16, 2018. The trainers then trained the field survey personnel, consisting of 94 enumerators and 357 interviewers, in six regions (Kisumu, Eldoret, Nakuru, Machakos, Nyeri, and Mombasa) from 19 to 23 March, 2018. The training was mainly conducted to assist with the hard copy questionnaire and computer-assisted personnel interview for data collection. In addition, both the interviewers and the supervisors were trained to conduct quality checks and send data to the servers.

After field deployment, the interviewers administered the questionnaire to every sampled household after obtaining consent. Interviewees were reminded that the information was voluntary and that they could terminate the interview at any stage. Monitoring was performed at all levels to ensure data quality; furthermore, both subject matter specialists and programmers were always available to deal with technical questions and device issues, respectively. There was an overall response rate of 95%; of the 37,500 sampled, we had complete household interviews for 33,286.

## Measurements

### Dependent Variable

Using the KHHEUS data collected for individuals and households in 2018, we included costs incurred for any outpatient healthcare in the 4 weeks prior to the survey. Here, outpatient healthcare means any medical procedures and services performed by a health facility and health providers (e.g., chemists and pharmacists) without the requirement of a stay in hospital.

These were collected based on registration cards, medicine/chemotherapy/vaccination, consultations, diagnosis tests (x-rays, lab, etc.), medical checkup, and dialysis. All expenses were calculated in Kenyan shillings (KSh) and then converted to American dollars (US$) using the mean exchange rate for the period set by the Central Bank of Kenya from January to December 2018 (1 US$ = 100.79 KSh).

### Independent Variables

To establish an association between total cost for outpatient care and its covariates, we selected variables that are commonly considered to predict healthcare cost and utilization. We included age, captured as a continuous variable; place of residence divided into urban and rural; and wealth index, divided into five different income groups (*poorest, poor, middle, rich, and richest*). Other selected variables included sex, captured as male or female; level of education, grouped into four categories (*none, primary, secondary, and post-secondary*; employment, captured as employed or unemployed; marital status, grouped into four categories (*single, married, separated, and divorced*; existence of a smoker in the household; and any member suffering from HIV, hypertension, cardiac problems, diabetes, mental health, cancer, TB, asthma, or any other respiratory problems. Employment was used as a proxy for the income of the household head.

We considered households headed only by a person aged 18 years and above. We calculated the total expenditure for people under outpatient care, as we were interested in estimating the healthcare utilization per household out of 11,130 households. In cases in which the respondent was not the head of the household, we considered the person who had the closest relationship with the household head as the head of the household.

Our response variable, that is, the total cost incurred for outpatient care, exhibited some characteristics that are of interest to this study related to users and non-users of outpatient services. Therefore, the response variable may have a discrete mass at zero (for non-users), continuous and right skewed (for users), with correlation for households that belong to the same cluster (county). To model such data, we adopted Tweedie distribution under generalized estimating equations (GEE) with an independent correlation structure.

Thereafter, we adopted the method of Hardin and Hilbe (41), which enabled us to check the best model fit using quasi-likelihood under the independence criterion (*QIC*_*u*_). *QIC*_*u*_ is a criterion proposed by Hardin and Hilbe (41) as an extension of the QIC proposed by Pan (42) for correlation structure selection, when no known structure of the data is known, or when there is no motivating scientific evidence of a particular correlation structure.

Our work has a predetermined correlation structure according to the guidelines provided by Hardin and Hilbe (41) of selecting the best correlation structure. Considering our panels, the differences in sizes of the number of subjects in each panel, and the fact that spending on healthcare among the panels may have weak correlation, we opted for the independence structure. In selecting the best subset of covariates, we evaluated the model with the lowest value for the *QIC*_*u*_ and the fewest number of covariates among competing models. We also evaluated the logarithmic and canonical links of the selected model.

### Statistical Methods

(EDM) has a probability density function that can be written as


(1) $$\begin{array}{l} p\left(y;\theta ,\phi \right)=bp\left(y,\phi \right)\text{exp }\left\{\frac{-d\left(y,\mu \right)}{2\phi }\right\} \end{array}$$

We assume that the cost for outpatient care during the survey period *N* follows a Poisson distribution with mean λ, such that if the household does not incur any cost, then *N* = 0. Finally, *Y* represents the total cost incurred by the household, which is represented as the Poisson sum of the gamma random variables, such that *Y* = *R*1+, …, +*RN*. Therefore, the resulting distribution may be called Poisson-gamma distribution.

Dunn and Smyth (43) showed that the probability density function for the Tweedie family can be represented as


(2) $$\begin{array}{l} \text{log}fp\left(y;\mu ,\phi \right)=\{\begin{array}{ll} -\lambda , & \text{for }y=0\\ -\frac{-y}{ϒ-\lambda -\text{log}y+\text{log}W\left(y,\phi ,p\right)}, & \text{for }y>0 \end{array} \end{array}$$

where ϒ = ϕ(*p* − 1)μ^*p*−1^, $\lambda =\frac{\mu^{2-p}}{\phi \left(2-p\right)}$, and *W* is an example identified by the Wright generalized Bessel function (44), which can be expressed as


(3) $$\begin{array}{l} W\left(y,\phi ,p\right)=\sum {j=1}^{\infty }\frac{y^{-j\alpha }{\left(p-1\right)}^{\alpha j}}{\phi^{j\left(1-\alpha \right){\left(2-p\right)}^{j}!\Gamma \left(-j\alpha \right)}} \end{array}$$

where


$$\alpha =\frac{\left(2-p\right)}{1-p}$$

with the mean of the Poisson-gamma given as μ and its variance given by


$$\text{Var}\left[y\right]=\phi \mu^{p}$$

### Approximating Tweedie Densities Using Saddle-Point Approximation

Various methods can be used to estimate a Tweedie density, including saddle-point, inversion, and interpolation (43, 45). In this study, we consider saddle-point approximation under the generalized linear model (GLM) to estimate the starting values for GEE.

A part of the density cannot be expressed in the closed part, *bp*(*y*, μ), as seen in equation 1, but can be replaced by a simple analytical expression, such that


(4) $$\begin{array}{l} p\left(y|\mu ,\phi \right)=\frac{1}{\sqrt{2\pi \phi y^{p}}}\text{exp }\left\{\frac{-d\left(y,\mu \right)}{2\phi }\right\}\left\{1+\omega \left(\phi \right)\right\} \end{array}$$

as ϕ → 0 for the Tweedie densities. The ratio is expressed as


(5) $$\begin{array}{l} \varsigma =bp\left(y,\phi \right)\sqrt{2\pi \phi y^{p}} \end{array}$$

such that


(6) $$\begin{array}{l} fp\left(y|\mu ,\phi \right)=\frac{1}{y}bp\left(1,\iota \right)\text{exp }\left\{\frac{-d\left(y,\mu \right)}{2\phi }\right\} \end{array}$$

where ι = ϕ^*p*−2^, such that the ratio of the density to the saddle-point is expressed as


(7) $$\begin{array}{l} \varsigma =bp\left(1,\iota \right)\sqrt{2\pi \iota } \end{array}$$

This shows that ς is a function of *p* and not μ, and is a function of *y* and ϕ through ι.

Using the Chebychev interpolation method (46), we can estimate any value of the parameter. The error is given by


(8) $$\begin{array}{l} f\left(x\right)-Pn\left(x\right)=\prod {1=0}^{n}\left(x-x_{i}\right)\frac{f^{\left(n+1\right)}\left(\varpi \left(x\right)\right)}{\left(n+1\right)!} \end{array}$$

such that we can reduce the interpolation error by choosing *xi*s to minimize.


(9) $$\begin{array}{l} \left||w\left(x\right)||=\text{max}x\in \left[a,b\right]|\prod {1=0}^{n}\left(x-x_{i}\right)\right| \end{array}$$

### Data Analysis

We investigated the following set of six models to understand the influence of covariates on predicting outpatient healthcare expenses in Kenya.

1. log μ = β_0_ + β_1_age + β_2_wealthIndex + β_3_maritalStatus + β_4_education
2. log μ = β_0_ + β_1_age + β_2_wealthIndex + β_3_education
3. log μ = β_0_ + β_1_age + β_2_wealthIndex + β_3_maritalStatus + β_4_sex
4. log μ = β_0_ + β_1_age + β_2_wealthIndex + β_3_maritalStatus + β_4_education + β_5_sex
5. log μ = β_0_ + β_1_age + β_2_wealthIndex
6. log μ = β_0_ + β_1_wealthIndex

*Model 6* represents the wealth index as a predictor of outpatient spending. The choice of its modeling lies in its *QIC*_*u*_ value against outpatient care spending, which is the lowest, as found by (32). *Model 5* controls for age and the wealth index. Age is found to have a lower *QIC*_*u*_ value than that of other covariates. Therefore, it is necessary to find its effect on the wealth index. *Model 4* controls for age, the wealth index, marital status, education, and sex of the household head. *Model 3* controls for age, the wealth index, marital status, and sex. *Model 2* controls for age, the wealth index, and education. Lastly, *Model 1* controls for age, the wealth index, marital status, and education.

In this study, we adopted a systematic approach to find the most suitable model, since it was not possible to investigate all possible outpatient cost models. First, a single predictor was developed and the *QIC*_*u*_ value was examined for each model. Second, models with the lowest *QIC*_*u*_ value were further examined. Third, predictors were added successively in order of importance, supported by the existing literature. Fourth, we chose the model that fits the data adequately after comparing the *QIC*_*u*_ values of the final models. We did not follow any specific order while modeling the covariates.

**Figure 1:**
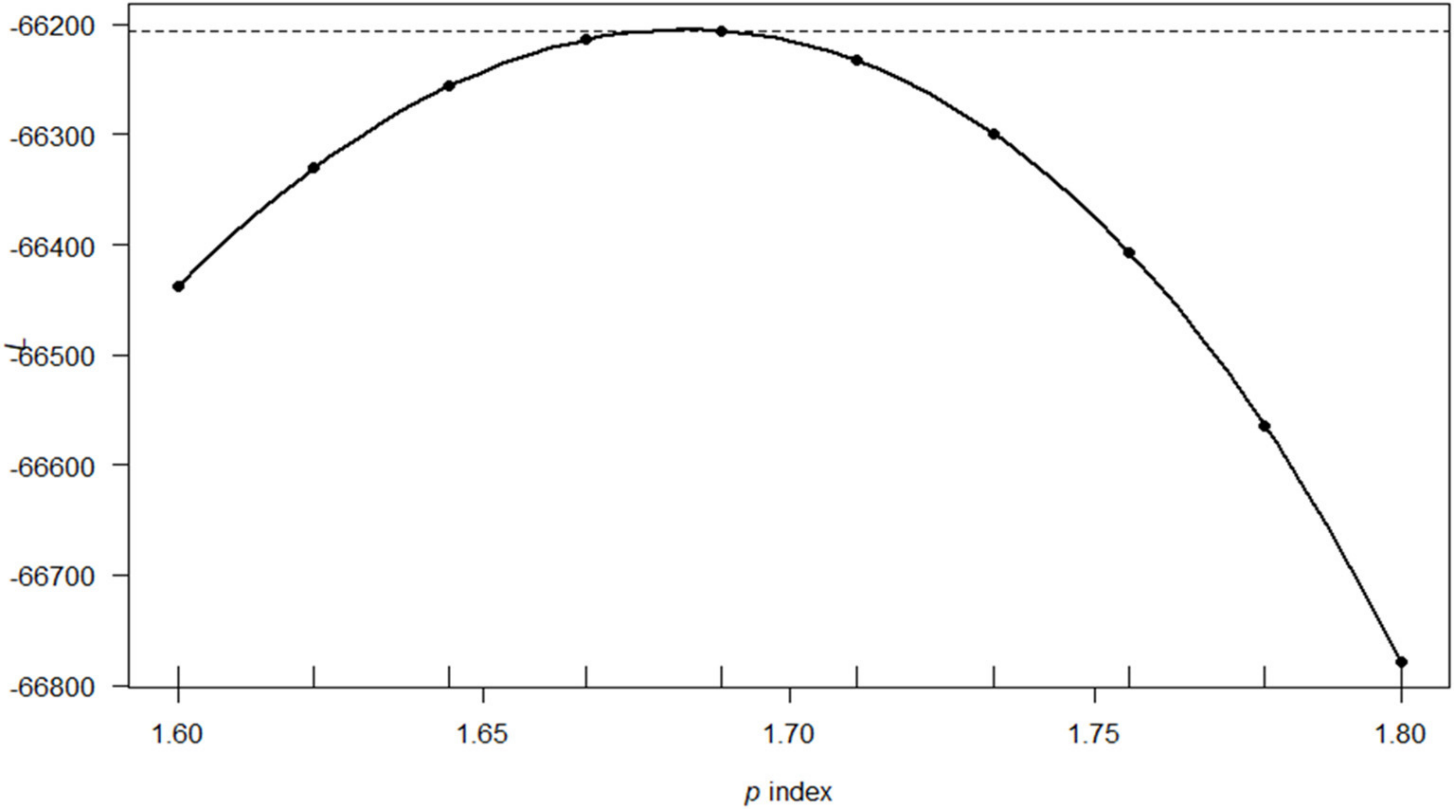
The profile log-likelihood plot for cost of outpatient care in Kenya using the Model 1 covariates. The solid line is a saddle-point approximation of the *P* index from the data with a value of 1.68 and estimated 95% confidence interval [1.67,1.69].

To fit a Tweedie GLM to the outpatient cost data, we estimated the variance power. This was achieved through the profile log-likelihood function of the maximum likelihood estimation (MLE) value corresponding to the most appropriate value of the variance function *p* with the respective 95% CI. Owing to computational difficulties associated with MLE, the variance parameter was obtained by maximizing the log-likelihood function. However, this was challenged by the presence of an infinity sum in the probability function and non-trivial restrictions on the power parameter space. Therefore, we fitted a cubic spline interpolation through these computed points, which was estimated as 1.68 through the software. Figure 1 shows the Tweedie profile with the estimated index parameter and the confidence interval for the best fitted model.

All statistical analyses were performed using the R programming language, version 3.6.3 (R Development Core Team, Vienna, Austria) (47). *P* < 0.05 indicates statistical significance.

## Results

A total of 11,130 households with heads above 18 years of age were studied. Socio-demographic characteristics of the respondents and their smoking status are shown in Table 1. The mean age of the respondents was 45.57 years with a standard deviation of 19.30 years. The majority of the households (62.79%) were male headed, married (68.60%), and residing in rural areas (63.18%). Meanwhile, the majority of the heads had up to primary education level (45.18%) or were unemployed (59.56%), and very few were smokers (7.96%). The wealth status of the households was evenly distributed across five quintiles from poorest to richest.

**Table 1:** Demographics of respondents (*N* = 11,130).

**Variable**	**Number**	**Percentage**
**Sex of head of household**		
Female	4,142	37.21
Male	6,988	62.79
**Relationship status**		
Single	1,251	11.24
Married	7,635	68.60
Separated	776	6.97
Divorced	1,468	13.19
**Residence**		
Rural	7,032	63.18
Urban	4,098	36.82
**Wealth Status**		
Poorest	2,075	18.64
Poor	2,202	19.78
Middle	2,469	22.18
Rich	2,559	22.99
Richest	1,825	16.40
**Employment status**		
Employed	4,612	41.44
Not-employed	6,518	58.56
**Education status**		
None	2,157	19.38
Primary	5,029	45.18
Secondary	2,883	25.90
Post-secondary	1,061	9.53
**Smoker**		
Yes	886	7.96
No	10,244	92.04
Age of household head (Mean ± SD) years	45.57 (19.30)	NA

Household members reporting any of health conditions selected for this study are shown in Table 2. There were double the number of people with hypertension (13.76%) compared to those with respiratory problems (7.48%). While the fewest cases were reported for cancer (0.67%), mental health (1.24%), and TB (1.58%), there were similar numbers of those suffering from HIV (4.19%), diabetes (4.20%), and asthma (5.83%).

**Table 2:** Any household member with the following conditions (*N* = 11,130).

**Variable**	**Number**	**Percentage**
**Hypertension**		
Yes	1,532	13.76
No	9,598	86.24
**Cardiac**		
Yes	232	2.08
No	10,898	97.92
**Diabetes**		
Yes	467	4.20
No	10,663	95.80
**Asthma**		
Yes	649	5.83
No	10,481	94.17
**Mental Health**		
Yes	138	1.24
No	10,992	98.76
**Cancer**		
Yes	75	0.67
No	11,055	99.33
**HIV**		
Yes	466	4.19
No	10,664	95.81
**Respiratory Illness**		
Yes	832	7.48
No	10,298	92.52
**TB**		
Yes	176	1.58
No	10,954	98.42

Table 3 summarizes the cost to non-users of outpatient healthcare and continuous costs for users in a household. Non-users do not spend money on outpatient care, while users spend different amounts. Summary statistics when both cases are analyzed together shows that users spend a minimum of 0.01 US$ and a maximum of 892.94 US$. The mean (SD) when users and non-users are analyzed together is 11.31 (32.07) US$ & 17.96 (38.90) US$, respectively, with skewness of 8.5 and 7.05, respectively, with the reference being the survey month.

**Table 3:** Summary of total costs of outpatient care incurred by households from the KHHEUS 2018.

**Statistic**	**Total cost ≥ 0 by the household**	**Total Cost > 0 by the household**
Minimum	0	0.01
Maximum	892.94	892.94
Mean	11.32	17.86
Median	1.69	6.35
Standard Deviation	32.07	38.90
Skewness	8.5	7.05
Characteristic of the skewness	Right skewed	Right skewed

*Statistics were recorded for the survey month total cost ≥ 0 US$ (all households) and survey month cost > 0 US$ (those who spend money on outpatient care only)*.

The resulting output after incorporating the *QIC*_*u*_ criterion, as explained in the six models, is shown in Table 4. The model with the lowest *QIC*_*u*_ was chosen as the best model.

**Table 4:** Different model outputs with calculated *QIC*_*u*_.

	**Model 1**			**Model 2**			**Model 3**			**Model 4**			**Model 5**			**Model 6**	
***QIC*_*u*_**	**976341.2**			976874			977759.3			985834			978755			982713.3	
Coefficient	$\hat{\beta }$	*p*		$\hat{\beta }$	*p*		$\hat{\beta }$	*p*		$\hat{\beta }$	*p*		$\hat{\beta }$	*p*		$\hat{\beta }$	*p*
(Intercept)	6.61	< 0.001		6.59	< 0.001		6.49	< 0.001		6.77	< 0.001		6.37	< 0.001		6.88	< 0.001
Age	0.01	< 0.001		0.01	< 0.001		0.01	< 0.001		0.01	< 0.001		0.01	< 0.001			
**Wealth index**																	
Ref (Poorest)																	
Poor	0.04	0.64		0.05	0.59		-0.01	0.87		0.04	0.68		0.00	0.98		−0.02	0.85
Middle	0.09	0.32		0.09	0.34		0.00	1.00		0.09	0.29		0.00	0.96		0.02	0.82
Rich	0.41	< 0.001		0.40	< 0.001		0.30	< 0.001		0.41	< 0.001		0.31	< 0.001		0.31	< 0.001
Richest	0.59	< 0.001		0.58	< 0.001		0.53	< 0.001		0.61	< 0.001		0.53	< 0.001		0.42	< 0.001
**Marital status**																	
Ref (Single)																	
Married	−0.04	0.63					0.00	1.00		−0.03	0.76						
Separated	−0.24	0.07					−0.15	0.25		−0.19	0.17						
Divorced	−0.22	0.07					−0.06	0.63		−0.12	0.35						
**Education**																	
Ref (None)																	
Primary	−0.25	< 0.001		−0.24	< 0.001					−0.27	< 0.001						
Secondary	-0.41	< 0.001		−0.38	< 0.001					−0.44	< 0.001						
Post secondary	−0.08	0.52		−0.05	0.70					−0.12	0.33						
**Sex**																	
Ref (Male)																	
Female							−0.16	< 0.001		−0.19	< 0.001						

*The model with the lowest QIC_u_ was selected as the best fitting model. In our case, Model 1 was selected as the most parsimonious model for predicting outpatient care cost among households in Kenya using the KHHEUS 2018*.

*Bold values shows the least of QICu for the best model*.

The best fitted model with the lowest *QIC*_*u*_ was Model 1. Its coefficients and covariates can be expressed as


$$\begin{array}{l} \log\mu =6.61+0.01\text{Age}+0.04\text{Poor}+0.09\text{Middle}+0.41\text{Rich}\\ \;+0.59\text{Richest}-0.04\text{Married}-0.24\text{Separated}\\ \;-0.22\text{Divorced}-0.25\text{Primary}-0.41\text{Secondary}\\ \;-0.08\text{Post-Secondary} \end{array}$$

where μ is the expected cost of outpatient care.

**Figure 2:**
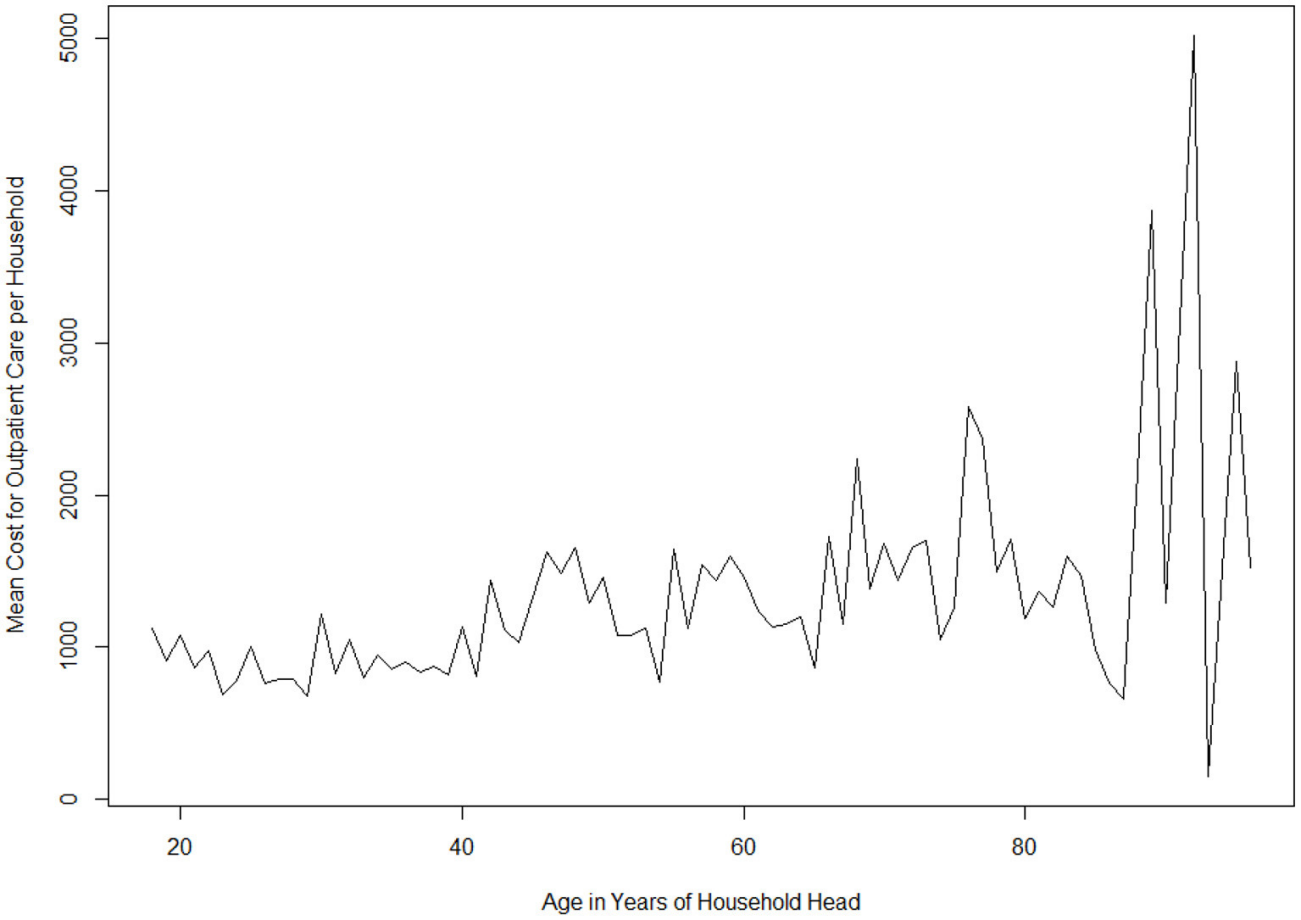
Variations of mean cost for outpatients by household head age.

Age of the household head was found to be a significant predictor of outpatient care expenses. A one-unit increase in age results in an increase in healthcare spending by a factor of 1.01 (*p* < *0.001*). The cost of outpatient care was found to change with age in a sinusoidal manner. Figure 2 shows the variation in total cost of outpatient expenses for households with respect to age of the household head during the survey period. A higher cost is associated with higher age of the household head.

Outpatient care costs increase across the wealth quantile with the rich and richest spending more, at 1.50 and 1.80, respectively, than the poorest. Household heads with primary and secondary levels of education spent less at 0.77 and 0.66, respectively, than those who never attended school. The results are significant at *p* = *0.05*.

We conducted additional applications on certain probabilities based on Dunn and Syth (43) to demonstrate the usefulness of Tweedie distribution in modeling cost for outpatient care. When 1 < *p* < 2, the Tweedie parameters (μ, *p*, ϕ) can be parameterized into Poisson and gamma parameters (λ, γ, α), which can be used to provide estimates for comparison with other outputs. This is given in the following equation


$$\begin{array}{l} \lambda =\mu^{\left(2-p\right)}/\phi \left(2-p\right) \end{array}$$

$$\begin{array}{l} \gamma =\phi \left(p-1\right)\mu^{\left(p-1\right)} \end{array}$$

$$\begin{array}{l} \alpha =\left(p-2\right)/\left(1-p\right) \end{array}$$

where λ is the average expenses per month, γ is the shape of the cost distribution when a household pays for outpatient care, and α γ is the mean expenses per month.

Considering our best fitted model, the parameter index *p* is 1.68, μ = exp(6.61)= 7.35 US$, and ϕ is 0.31 US$. Reparameterizing to gamma and Poisson yields the predicted mean cost expenditure per month, calculated as


$$\lambda =\frac{7.35^{\left(2-1.68\right)}}{0.31\left(2-1.68\right)}=0.84$$

and


$$\gamma =0.31\left(1.68-1\right)7.35^{\left(1.68-1\right)}=19.09$$

finally


$$\alpha =\frac{1.68-2}{1-1.68}=0.47$$

The mean expenditure per household on outpatient care is αγ = 0.47 * 19.09 = 8.97 US$.

Following Dunn and Smyth (43), the probability of incurring zero cost on outpatient care by households (i.e., the probability of not seeking outpatient care) is given by


(10) $$\begin{array}{l} \text{Pr}\left(Y=0\right)=\text{exp}\left(-\lambda \right)=\text{exp }\left[-\frac{\mu^{2-p}}{\phi \left(2-p\right)}\right] \end{array}$$

such that, the probability of zero outpatient care is given by exp(−0.84) = 0.43, meaning that 43% of households did not spend on outpatient care in any given month. Therefore, 57% of households spent money on outpatient costs.

Finally, we investigated the deviance obtained from using the two different link functions, as shown in Table 5. Using the logarithmic link was appropriate, since it had lower deviance than the default canonical link function.

**Table 5:** The residual deviance and degrees of freedom for a Tweedie GLM with differing link functions using Model 1 covariates.

**Link function**	**Deviance**	**DF**
Logarithm	404663.6	11118
Canonical	404872.7	11118

## Discussion and Conclusion

This study analyzed the responses of members of households who attended outpatient facilities in Kenya in 2018; it investigated the best predictors for outpatient care in correlation with the household head characteristics. The best predictors were obtained from the most parsimonious model with the lowest *QIC*_*u*_. Three key findings emerged: age of the household head, education, and the wealth index were associated with spending on outpatient care.

Households headed by older members were associated with higher spending. This can partly be explained by the fact that higher age could signify (1) the aged suffering from chronic and serious illnesses that are expensive to treat, (2) more members in the household needing these services, and (3) higher incomes to pay for a service. This finding corroborates previous evidence showing that out-of-pocket spending for outpatient care increased correlatively with age in Kenya (40). Additional evidence showed an increase in spending on healthcare among the aged in emerging economies (48). Thus, the burden of healthcare is higher in households headed by older people. Another insight requiring further analysis is that households headed by older people were also the respondents.

The rich and richest wealth quintiles spent more on outpatient services than the poorest did. Similar results have been reported in prior studies, where poor utility among the poor was observed in Zimbabwe (49), south west Ethiopia (50), Brazil (51), and in rural areas of Kenya (52). Spending on outpatient healthcare could be influenced by financial health, which leads to choices about where to seek care. Therefore, it is not surprising that the rich and richest households reported higher costs. This is possibly because the rich mostly seek care in private facilities (7), which are expensive. Further evidence published in a technical report on the findings of the KHHEUS survey showed that per capita expenditure increased relative to wealth (per capita expenditure for the rich was 23.58 US$, that for the richest was 32.11 US$, and that for the poorest was 12.01 US$) (40).

Households that have heads with secondary and primary education spent less on outpatient care than did those who never went to school. Previous studies have found that per capita expenditure on outpatient care by group was 15.20 US$ for those with primary education, 20.34 US$ for those with secondary education, and 27.80 US$ for those who had never attended school.

There is emerging evidence of a negative correlation between education and self-medication (53). Self-medication could have lower costs, as it is mostly associated with drug purchase over the counter (54) for less complicated cases, such as headaches and abdominal discomfort (55). Thus, those without education may rely on facilities to diagnose their symptoms, thereby incurring more expenses. A similar observation was observed in Vietnam, that increased education reduced outpatient healthcare utilization (56). It has been argued that an increase in education could have positive impacts on health-related outcomes, such as low risks of illnesses and healthier habits (57).

The results have significant practical implications for Kenya, where much debate revolves around cushioning the public from catastrophic spending. Most of the literature in Kenya on determinants of catastrophic spending have critically considered cash spending on both inpatient and outpatient care. For example, the fourth round of the KHHEUS study found that four times more out-of-pocket spending was witnessed in outpatient than inpatient care (0.929 billion US$ against 0.253 billion US$, respectively) (40).

There has been consistent effort by the government of Kenya and development institutions, such as the WB, to reduce poverty among citizens, so as to raise their socio-economic status and free up household income to spend on healthcare (58). The inability to pay the fees charged at a health facility is a hindrance to Kenyans seeking care (59).

Outpatient spending has been a major source of catastrophic spending in Kenya, and mostly has been paid from household savings and income (60). Similar results have been recorded elsewhere in developing countries, such as India (61) and Nepal (62). In Kenya, the debate centers on whether to improve public facilities to make them more desirable choices for healthcare treatment, or to provide insurance to households so that members can seek care in either public or private facilities (63). Therefore, policies targeting UHC, especially healthcare affordability, should continue to be implemented, as this would ease the burden of spending on households and direct such resources elsewhere to improve living standards.

This study has a number of limitations. Age is an endogenous variable, and thus, its increase does not necessarily point to severe disease, but could possibly be due to financial freedom that comes with age. A clear analysis stratifying age with wealth is necessary to decode this finding. However, since this work is based on determining the overall best predictors of outpatient spending, it is beyond the scope of this study. In addition, this work focused on spending at the household level; it is possible that most household spending was on the aged. An individual analysis on specific age groups could help shed light on this scenario.

## Data Availability Statement

The raw data supporting the conclusions of this article will be made available by the authors, without undue reservation.

## R Code for Replicating Our Results

We analyzed this study using the R programming language. The R code is archived at GitHub and can be accessed using the link https://github.com/samwenda/Tweedie-and-Indpedent-correlation.

## Author Contributions

All authors listed have made a substantial, direct and intellectual contribution to the work, and approved it for publication.

## Conflict of Interest

The authors declare that the research was conducted in the absence of any commercial or financial relationships that could be construed as a potential conflict of interest.

## Publisher's Note

All claims expressed in this article are solely those of the authors and do not necessarily represent those of their affiliated organizations, or those of the publisher, the editors and the reviewers. Any product that may be evaluated in this article, or claim that may be made by its manufacturer, is not guaranteed or endorsed by the publisher.

## References

[1] World Bank. Kenya Among the Fastest Growing Economies in Africa. World Bank (2015).

[2] Kukla M McKay N Rheingans R Harman J Schumacher J Kotloff KL. The effect of costs on Kenyan households-demand for medical care: why time and distance matter. Health Policy Plann. (2017) 32:1397–406. 10.1093/heapol/czx120 29036378

[3] O'Neill A. Kenya: National Debt in Relation to Gross Domestic Product (GDP) from 2016 to 2026. Nairobi: Statistica Company (2021).

[4] Huaxia. Kenya's Unemployment Rate Doubles to 10.4 PCT in Q2 Due to COVID-19. XHINUANET (2020).

[5] Pezzulo C Hornby GM Sorichetta A Gaughan AE Linard C Bird TJ. Sub-national mapping of population pyramids and dependency ratios in Africa and Asia. Sci Data. (2017) 4:170089. 10.1038/sdata.2017.89 28722706 PMC5516541

[6] Kimathi L. Challes of the devolved health sector in Kenya: teething problems or systemic contradictions? Africa Dev. (2017) 42:55–77. 34020287

[7] MoH. Is Kenya Allocating Enough Funds for Healthcare? (2021).

[8] McIntyre D Thiede M Dahlgren G Whitehead M. What are the economic consequences for households of illness and of paying for health care in low- and middle-income country contexts? Soc *Sci Med*. (2006). 62:858–65. 10.1016/j.socscimed.2005.07.001 16099574

[9] Rabarison KM Bish CL Massoudi MS Giles WH. Economic evaluation enhances public health decision making. Front Public Health. (2015) 3:164. 10.3389/fpubh.2015.00164 26157792 PMC4478374

[10] Grepin KA Pinkstaff CB Shroff ZC Ghaffar A. Donor funding health policy and systems research in low- and middle-income countries: how much, from where and to whom. Health Res Policy Syst. (2017) 15:68. 10.1186/s12961-017-0224-6 28854946 PMC5577666

[11] Robertson L Skelly C Phillips D. Making hard choices in local public health spending with a cost-benefit analysis approach. Front Public Health. (2019) 7:147. 10.3389/fpubh.2019.00147 31316957 PMC6609906

[12] Liu H Dai W. An empirical study on the benefits equity of the medical security policy: the China Health and Nutrition Survey (CHNS). Int J Environ Res Public Health. (2020) 17:1203. 10.3390/ijerph17041203 32069973 PMC7068417

[13] Li L Jiang J Xiang L Wang X Zeng L Zhong Z. Impact of critical illness insurance on the burden of high-cost rural residents in central China: an interrupted time series study. Int J Environ Res Public Health. (2019) 16:3528. 10.3390/ijerph16193528 31547215 PMC6801576

[14] Jing R Xu T Lai X Mahmoudi E Fang H. Technical efficiency of public and private hospitals in Beijing, China: a comparative study. Int J Environ Res Public Health. (2020) 17:82. 10.3390/ijerph17010082 31861922 PMC6981764

[15] Lee WY Shaw I. The impact of out-of-pocket payments on health care inequity: the case of national health insurance in South Korea. Int J Environ Res Public Health. (2014) 11:7304–18. 10.3390/ijerph110707304 25046630 PMC4113877

[16] Li A Shi Y Yang X Wang Z. Effect of critical illness insurance on household catastrophic health expenditure: the latest evidence from the National Health Service Survey in China. Int J Environ Res Public Health. (2019) 16:5086. 10.3390/ijerph16245086 31847072 PMC6950570

[17] Kato R Okada M. Can financial support reduce suicide mortality rates? Int J Environ Res Public Health. (2019) 16:4797. 10.3390/ijerph16234797 31795379 PMC6926693

[18] Oxford Business Group. New Health Care Initiatives in Kenya to Increase Access and Quality. Oxford Business Group (2020).

[19] Mwenda V Mwangi M Nyanjau L Gichu M Kyobutungi C Kibachio J. Dietary risk factors for non-communicable diseases in Kenya: findings of the STEPS survey, (2015). BMC Public Health. (2018) 18:1218. 10.1186/s12889-018-6060-y 30400904 PMC6219002

[20] Aluga MA. Coronavirus Disease 2019 (COVID-19) in Kenya: Preparedness, response and transmissibility. J Microbiol Immunol Infect. (2020). 53:671–3. 10.1016/j.jmii.2020.04.011 32331980 PMC7167550

[21] Janssens W Pradhan M de Groot R Sidze E Donfouet HPP Abajobir A. The short-term economic effects of COVID-19 on low-income households in rural Kenya: an analysis using weekly financial household data. World Dev. (2021) 138:105280. 10.1016/j.worlddev.2020.105280

[22] Chuma J Maina T. Catastrophic health care spending and impoverishment in Kenya. BMC Health Serv Res. (2012) 12:413. 10.1186/1472-6963-12-413 23170770 PMC3561146

[23] Barasa EW Maina T Ravishankar N. Assessing the impoverishing effects, and factors associated with the incidence of catastrophic health care payments in Kenya. Int J Equity Health. (2017) 16:31. 10.1186/s12939-017-0526-x 28166779 PMC5294805

[24] Mbau R Kabia E Honda A Hanson K Barasa E. Examining purchasing reforms towards universal health coverage by the National Hospital Insurance Fund in Kenya. Int J Equity Health. (2020) 19:19. 10.1186/s12939-019-1116-x 32013955 PMC6998279

[25] Barasa E Rogo K Mwaura N Chuma J. Kenya National Hospital Insurance Fund reforms: implications and lessons for universal health coverage. Health Syst Reforms. (2018) 4:346–61. 10.1080/23288604.2018.1513267 30398396 PMC7116659

[26] Obare V Brolan CE Hill PS. Indicators for universal health coverage: can Kenya comply with the proposed post-2015 monitoring recommendations? Int J Equity Health. (2014) 13:123. 10.1186/s12939-014-0123-1 25532714 PMC4296682

[27] Okech TC Lelegwe SL. Analysis of universal health coverage and equity on health care in Kenya. Glob J Health Sci. (2015) 8:218–27. 10.5539/gjhs.v8n7p218 26925910 PMC4965667

[28] Barasa E Nguhiu P McIntyre D. Measuring progress towards Sustainable Development Goal 3.8 on universal health coverage in Kenya. BMJ Global Health. (2018) 3:1–12. 10.1136/bmjgh-2018-000904 29989036 PMC6035501

[29] Salari P Di Giorgio L Ilinca S Chuma J. The catastrophic and impoverishing effects of out-of-pocket healthcare payments in Kenya, (2018). BMJ Global Health. (2019) 4:1–13. 10.1136/bmjgh-2019-001809 31803510 PMC6882550

[30] Kimani DN. Out-of-Pocket Health Expenditures and Household Poverty: Evidence from Kenya. University of Nairobi (2014).

[31] Ilinca S Di Giorgio L Salari P Chuma J. Socio-economic inequality and inequity in use of health care services in Kenya: evidence from the fourth Kenya household health expenditure and utilization survey. Int J Equity Health. (2019) 18:196. 10.1186/s12939-019-1106-z 31849334 PMC6918604

[32] Awiti JO. Poverty and health care demand in Kenya. BMC Health Serv Res. (2014) 14:560. 10.1186/s12913-014-0560-y 25416417 PMC4243287

[33] Umar N Litaker D Schaarschmidt ML Peitsch WK Schmieder A Terris DD. Outcomes associated with matching patients' treatment preferences to physicians' recommendations: study methodology. BMC Health Serv Res. (2012) 12:1. 10.1186/1472-6963-12-1 22214259 PMC3276415

[34] Mwabu GM. Nonmonetary factors in the household choice of medical facilities. Econ Dev Cult Change. (1989) 37:383–92. 10.1086/451728

[35] Ensor T Cooper S. Overcoming barriers to health service access: influencing the demand side. Health Policy Plann. (2004) 19:69–79. 10.1093/heapol/czh009 14982885

[36] Vallieres F Hansen A McAuliffe E Cassidy EL Owora P Kappler S. Head of household education level as a factor influencing whether delivery takes place in the presence of a skilled birth attendant in Busia, Uganda: a cross-sectional household study. BMC Pregnancy Childbirth. (2013) 13:48. 10.1186/1471-2393-13-48 23433253 PMC3623753

[37] Bradshaw S. Women's decision-making in rural and urban households in Nicaragua: the influence of income and ideology. Environ Urban. (2013) 25:81–94. 10.1177/0956247813477361

[38] Acharya DR Bell JS Simkhada P van Teijlingen ER Regmi PR. Women's autonomy in household decision-making: a demographic study in Nepal. Reproduct Health. (2010) 7:15. 10.1186/1742-4755-7-15 20630107 PMC2914657

[39] Posel DR. Who are the heads of household, what do they do, and is the concept of headship useful? An analysis of headship in South Africa. Dev South Africa. (2001) 18:651–70. 10.1080/03768350120097487

[40] MoH. Kenya Household Health Expenditure and Utilization Survey. Ministry of Health (2018).

[41] Hardin JW. Generalized Estimating Equations. Hardin; Boca Raton, FL: CRC Press (2013). 10.1002/9781118445112.stat06428

[42] Pan W. Akaike's information criterion in generalized estimating equations. Biometrics. (2001) 57:120–5. 10.1111/j.0006-341X.2001.00120.x 11252586

[43] Dunn PK Smyth GK. Series evaluation of Tweedie exponential dispersion model densities. Stat Comput. (2005) 15:267–80. 10.1007/s11222-005-4070-y

[44] Wright EM. On the coefficients of power series having exponential singularities. J Lond Math Soc. (1933) 8:71–9. 10.1112/jlms/s1-8.1.71

[45] Dunn PK. Tweedie: Evaluation of Tweedie Exponential Family Models. R package version 2.3.0 (2017).

[46] Salzer HE. Chebyshev interpolation and quadrature formulas of very high degree. Commun ACM. (1969) 12:271. 10.1145/362946.362980

[47] R Core Team. R: A Language and Environment for Statistical Computing. Vienna (2017). Available online at: http://www.R-project.org/

[48] Zhou L Ampon-Wireko S Asante Antwi H Xu X Salman M Antwi MO. An empirical study on the determinants of health care expenses in emerging economies. BMC Health Serv Res. (2020) 20:774. 10.1186/s12913-020-05414-z 32838767 PMC7444191

[49] Kevany S Murima O Singh B Hlubinka D Kulich M Morin SF. Socio-economic status and health care utilization in rural Zimbabwe: findings from Project Accept (HPTN 043). J Public Health Africa. (2012) 3:e13. 10.4081/jphia.2012.e13 22962629 PMC3436598

[50] Girma F Jira C Girma B. Health services utilization and associated factors in Jimma zone, South west Ethiopia. Ethiopian J Health Sci. (2011) 21:85–94. 22435012 PMC3275873

[51] O'Donnell O. Access to health care in developing countries: breaking down demand side barriers. Cadernos de Saúde Pública. (2007) 23:2820–34. 10.1590/S0102-311X2007001200003 18157324

[52] Ngugi AK Agoi F Mahoney MR Lakhani A Mang'ong'o D Nderitu E. Utilization of health services in a resource-limited rural area in Kenya: prevalence and associated household-level factors. PLoS ONE. (2017) 12:e0172728. 10.1371/journal.pone.0172728 28241032 PMC5328402

[53] Shaghaghi A Asadi M Allahverdipour H. Predictors of self-medication behavior: a systematic review. Iran J Public Health. (2014) 43:136–46. 26060736 PMC4450680

[54] Yadav S Rawal G. Self-medication practice in low income countries. Int J Pharmaceut Chem Anal. (2015) 2:139–42. 32932630

[55] Mekuria AB Birru EM Tesfa MT Geta M Kifle ZD Amare T. Prevalence and predictors of self-medication practice among teachers' education training college students in Amhara Region, Ethiopia: a cross-sectional study. Front Pharmacol. (2021) 11:2457. 10.3389/fphar.2020.593764 33603664 PMC7884827

[56] Dang T. Do the more educated utilize more health care services? Evidence from Vietnam using a regression discontinuity design. Int J Health Econ Manage. (2018) 18:277–99. 10.1007/s10754-018-9233-4 29322279

[57] Grossman M. The correlation between health and schooling. In: Terleckyj NE editor. Household Production and Consumption. NBER (1976). p. 147–224.

[58] Pape U Mejia-Mantilla C. More than just growth: accelerating poverty reduction in Kenya (2019).

[59] Muriithi MK. The determinants of health-seeking behavior in a Nairobi Slum, Kenya. Eur Sci J. (2013) 9:151–64.

[60] Berman P Ahuja R Bhandari L. The impoverishing effect of healthcare payments in India: new methodology and findings. Econ Political Weekly. (2010) 45:65–71.

[61] Joe W. Distressed financing of household out-of-pocket health care payments in India: incidence and correlates. Health Policy Plann. (2015) 30:728–41. 10.1093/heapol/czu050 24966294

[62] Adhikari SR Maskay NM Sharma BP. Paying for hospital-based care of Kala-azar in Nepal: assessing catastrophic, impoverishment and economic consequences. Health Policy Plann. (2009). 24:129–39. 10.1093/heapol/czn052 19181674

[63] Gupta I Chowdhury S Prinja S Trivedi M. Out-of-pocket spending on out-patient care in India: assessment and options based on results from a district level survey. PLoS ONE. (2016) 11:e0166775. 10.1371/journal.pone.0166775 27861559 PMC5115797

